# Contextuality, Complementarity, Signaling, and Bell Tests

**DOI:** 10.3390/e24101380

**Published:** 2022-09-28

**Authors:** Andrei Khrennikov

**Affiliations:** International Center for Mathematical Modeling in Physics and Cognitive Sciences, Linnaeus University, SE-351 95 Växjö, Sweden; andrei.khrennikov@lnu.se

**Keywords:** contextuality, complementarity, Bell inequalities, quantum nonlocality, joint probability distribution, Växjö model for contextual probability, signaling, contextuality by default

## Abstract

This is a review devoted to the complementarity–contextuality interplay with connection to the Bell inequalities. Starting the discussion with complementarity, I point to contextuality as its seed. *Bohr contextuality* is the dependence of an observable’s outcome on the experimental context; on the system–apparatus interaction. Probabilistically, complementarity means that the *joint probability distribution* (JPD) does not exist. Instead of the JPD, one has to operate with contextual probabilities. The Bell inequalities are interpreted as the statistical tests of contextuality, and hence, incompatibility. For context-dependent probabilities, these inequalities may be violated. I stress that contextuality tested by the Bell inequalities is the so-called *joint measurement contextuality* (JMC), the special case of Bohr’s contextuality. Then, I examine the role of signaling (marginal inconsistency). In QM, signaling can be considered as an experimental artifact. However, often, experimental data have signaling patterns. I discuss possible sources of signaling—for example, dependence of the state preparation on measurement settings. In principle, one can extract the measure of “pure contextuality” from data shadowed by signaling. This theory is known as *contextuality by default* (CbD). It leads to inequalities with an additional term quantifying signaling: Bell–Dzhafarov–Kujala inequalities.

## 1. Introduction

This is a review devoted to the interplay of notions of contextuality and complementarity as the interpretational basis of the violation of the Bell inequalities [1,2,3]. I make essential efforts to clarify and logically structure Bohr’s views [4] on contextuality and contextuality’s crucial role in the derivation of the complementarity principle [5,6,7,8,9,10,11,12,13,14,15,16] (see also [17,18]). In fact, in Bohr’s writings these two notions are really inseparable. I recommend to the reader the books of Plotnitsky and Jaeger [19,20,21,22] clarifying Bohr’s views on complementarity and contextuality. Bohr did not use the notion of contextuality. He wrote about experimental conditions. However, in the modern terminology he appealed to the contextuality of quantum measurements. I remark that at the beginning Bell did not use this terminology. This notion was invented in QM by Beltrametti and Cassinelli [23].

In philosophic terms, Bohr’s contextuality means rejection of “*naive realism*”; for Bohr the outcomes of quantum measurements cannot be treated as the objective properties of a system under observation. These values cannot be assigned to a system before a measurement, with the exception of a special system’s states–the eigenstates of observables. However, I do not like to operate with the notion of realism when including the EPR elements of reality. I leave this field for philosophers who have been working on it during the last two thousands years. Instead, I will work with the notion of Bohr’s contextuality which is formulated in the heuristically clear physical terms–the interaction between a system and a measurement device. I do not want to operate with the notion of *local realism* either. This is an ambiguous notion. (However, this is just author’s personal viewpoint.) At the least, one has to split local realism into two components, realism and locality, and then analyze them separately. I briefly discuss this notion and its components in Appendix A.

In this review I do not try to cover all approaches to contextuality; in particular, I do not discuss the Kochen–Specker theorem and the corresponding contextuality (see the recent review of Svozil [24] for a description of the diversity of the views on contextuality).

Starting with mentioning the Bohr principle of complementarity, also known as “wave–particle duality”, I analyze the notion of contextuality. The latter is understood very generally, as the irreducible dependence of observable’s outcome on the experimental context. Thus, the outcomes of quantum observables are not the objective properties of systems. They are generated in the complex process of interaction between a system and a measurement device. In fact, “*Bohr contextuality*” is the seed of complementarity, the existence of incompatible observables [5,6,7,8,9,10,11,12,13,14,15,16].

In probabilistic terms, incompatibility means that JPD does not exist. Instead of the JPD, one has to operate with a family of probability distributions depending on experimental contexts as in the *the Växjö model* for contextual probability theory [25,26,27,28,29,30,31,32,33,34,35,36]. This model generalizes the notion of conditional probability from classical probability (CP) theory. In some cases, the contextual probability update can be represented via the state update of the projection type represented in the complex Hilbert space [26,27,28,29,34,37,38]. Additionally, of course, the probability update of quantum theory can be easily realized as an update of contextual probability. The update machinery is formalized via introduction of special contexts corresponding to the outcomes of observables [25,26,27,28,29,30,31,32,33,34,35,36].

I continue to analyze the probabilistic structure of QM by considering the Bell inequalities and concentrating on the CHSH inequality [39] and Fine’s theorem [40]. This theorem connects the Bell inequality with the existence of the JPD for four observables involved in the Bohm–Bell experiment—in fact, the group of four separate experiments for the pairwise measurements for some pairs of these observables. I use the Fine theorem as the bridge to the contextual interpretation of the Bell-type inequalities. For context-dependent probabilities in the absence of a JPD unifying them, these inequalities can be violated [34]. I point out that the contextuality tested by the Bell inequalities is the so-called *joint measurement contextuality* (JMC) [2] (and Section 2.3)—the very special case of Bohr’s contextuality. I stress that consideration of the JMC is dominating within the quantum studies of contextuality. On one hand, this simplifies the picture; on the other hand, by reducing Bohr’s contextuality to JMC, people miss the general contextual perspective as it was established by Bohr at the very beginning of QM.

Some authors even define contextuality directly as the violation of some Bell inequality (see, e.g., [41] and references therein). I call such type of contextuality *Bell contextuality.* However, Bell by himself invented contextuality [2] as JMC, and then he pointed out that JMC can serve as a source of “Bell contextuality”.

I remark that originally Bell explained the violation of his inequality by Einsteinian nonlocality [42]: “spooky action at a distance”. In the article [2], Bell discussed contextuality in the JMC form in connection with nonlocality (see also the related papers of Gudder [43,44,45] and Shimony [46,47]). However, JMC per se cannot clarify the origin of Einsteinian nonlocality. In Bell’s discussion [2], JMC looks even more mystical than nonlocality. Consideration of JMC as the special case of Bohr contextuality and connecting it with incompatibility demystifies JMC. Additionally, by highlighting the role of incompatibility, the debate on the meaning of the Bell-type inequalities turns to the very basics of QM, to Bohr’s complementarity principle and the existence of incompatible observables. *The Bell inequalities are interpreted as the special tests of contextuality, and hence, incompatibility* [6,7]. Coupling contextuality and incompatibility is basic in our treatment of the Bell inequalities. This review continues the line of articles—*“getting rid off nonlocality from quantum physics"* [6,7,8,9] (see also [48,49,50,51,52,53,54,55,56,57,58,59,60,61,62,63,64,65]).

I also examine *signaling*, which may be better to call *marginal inconsistency*, by following the line of research presented in articles of Adenier and Khrennikov [66,67,68,69,70,71]. Typically, its role in discussions on the Bell inequalities is not highlighted. In contrast to the majority of authors, I take very seriously complications related to the presence signaling patterns in experimental statistical data [67]. It must be noted that the terminology “signaling” is quite ambiguous, since in fact “signaling” is defined not in terms of signals propagating in physical space-time, but in a purely probabilistic framework, as non-coincidental marginal probability distributions corresponding to joint measurements of an observable *a* with other observables which are compatible with it.

In QM, signaling can be considered as an experimental artifact—theoretically there should be no signaling. However, often experimental data have signaling patterns which are statistically non-negligible [67,72,73,74,75,76]. I discuss possible sources of signaling, both in the theoretical and experimental frameworks. In particular, I point to dependence of the state preparation procedure on settings of measurement devices as a signaling source (cf. [72,73,77]): the standard source state generation is supplemented with an additional state modification which is setting-dependent.

I emphasize that in the studies on interrelation between classical and quantum physics, signaling cannot be ignored. The presence of signaling in the experimental statistical data per se means that such data deviate from the theoretical prediction of QM. In accordance with the general methodology for random experiments, researchers have to check statistical significance of the signaling hypothesis. If signaling is statistically significant, then there is no need to check whether some Bell inequality is violated or not. In the presence of signaling approaching the high level of the violation of, e.g., the CHSH inequality, it is totally meaningless. Even tremendous efforts to close all possible loopholes are meaningless if data suffer from statistically non-negligible signaling.

I remark that, as was recently found by Dzhafarov et al. [78,79,80,81,82,83,84,85], one can extract the measure of pure contextuality even from statistical data with statistically significant signaling. This theory is known as *contextuality by default* (CbD). Foundationally, it is based on a representation of observables by context-dependent random variables (similarly to the Växjö model). Mathematically, CbD is based on the *coupling technique* of CP [86,87]. CbD with the mathematical technique from CP leads to the Bell inequalities with the additional term quantifying the level of signaling. I call such inequalities the *Bell–Dzhafarov–Kujala inequalities* (BDK). In this review, I am concentrated on the CHSH-BDK inequality.

Mathematically, CbD can be considered as a part of the project on the CP treatment of the Bell inequalities and contextuality. Another part of this project was presented in [88,89,90,91], where *quantum probabilities were treated as classical conditional probabilities* with conditioning with respect to the selection of experimental settings (cf. with Koopman [92] and Ballentine [50,93,94,95,96]). This is the good place to mention the CP-based tomographic approach to QM which was developed by Vladimir Man’ko and coauthors [97,98,99,100]. I also point to the articles [84,101] for a debate on the perspectives of CP use in contextual modeling (without direct connection with QM).

The author also would like to inform physicists that nowadays quantum theory, its methodology and its mathematical formalism, are widely applied outside of physics, to cognition, psychology, decision making, social and political sciences, economics and finance (see, e.g., monographs [102,103,104,105,106,107,108] and references in them). The author called this kind scientific research *quantum-like modeling*, and this terminology became widespread. In particular, contextuality based on quantum studies attracted a lot of attention, especially in cognitive psychology and decision making, including the Bell tests [106,109,110,111,112,113,114,115,116]. One of the specialties of such studies is the presence of signaling patterns in statistical data collected in all experiments which were done up to now [113]. Here the BDK-inequalities are especially useful [115,116].

In this review I discuss mainly the CHSH inequality. This was motivated by two reasons, experimental and theoretical ones. The basic experiments have been done for this inequality [72,73,75,117,118] (with some very important exceptions [74,119]; see also [76]). The mathematical structure of this inequality makes it possible to establish the straightforward coupling with incompatibility expressed mathematically in the form of commutators [6] (Section 6). From the author’s viewpoint, the original Bell inequality derived under the assumption of the prefect correlations deserves more attention, both theoretically and experimentally; some steps in this direction were made in the following studies [120,121,122].

In this review I am concentrated only on the Bohr contextuality and its “derivatives”, JMC and Bell contextuality; nor do I discuss hidden variables theory. The latter may be surprising, since from the beginning the Bell inequalities were derived in the hidden variables framework. However, I treat these inequalities as statistical tests of incompatibility. In the presence of incompatible observables, it is meaningless to discuss theories with hidden variables, at least theories in which hidden variables are straightforwardly connected with the outcomes of observables, as was done by Bell and his followers. Already, De Broglie pointed out that such theories have no physical meaning.

In principle, one can consider subquantum models, but variables of such models are only indirectly coupled to outcomes of quantum observables. The latter viewpoint was advertised by Schrödinger [123,124], who in turn followed the works fo Hertz [125] and Boltzmann [126,127] (see also [128,129]). One of such subquantum theories was developed in the series of the author’s works on the emergence of QM from classical random field theory [130].

## 2. Preliminary Discussion

### 2.1. Forgotten Contribution of Bohr to Contextuality Theory

Contextuality is one of the hottest topics of modern quantum physics, both theoretical and experimental. During the recent 20 years, it was discussed in numerous papers published in top physics journals. An unfortunate part of these discussions is that from the very beginning, contextuality (JMC, Section 2.3) was coupled to the issue of nonlocality. This was Bell’s intention in his analysis of the possible seeds of the violation of the Bell type inequalities [2].

Surprisingly, Bell never mentioned general contextuality which we call “*Bohr contextuality*”. The latter has no straightforward coupling to the Bell inequalities; it is closely related to the notion of the incompatibility of observables—the *Bohr principle of complementarity.* What is even more surprising is that Shimony, who was one of authorities in quantum foundations by commenting [46,47] on Bell’s article [2], also failed to mention the Bohr principle of complementarity and its contextual dimension.

One of the explanations for this astonishing situation in quantum foundations is that Bohr presented his ideas in a vague way; moreover, he changed his vague formulations a few times on different occasions. In this section, I briefly present Bohr’s ideas about the contextuality of quantum measurements and its role in his formulation of the complementarity principle (see [5,6,7,8,9,10,11,12,13,14,15,16] for detailed presentations). Then, I move to the Bell inequalities. Coupling these inequalities with Bohr’s contextuality and complementarity highlights the role of the incompatibility of quantum observables. It shows that we can operate with the Bell inequalities without mentioning the ambiguous notion of quantum nonlocality (spooky action at a distance).

### 2.2. What Does Contextuality Mean?

In this situation of so many researchers writing and speaking about quantum contextuality, one should be sure that this notion is well defined and its *physical interpretation* is clear and well known. In fact, before he started to think about the meaning of contextuality, the author of this paper was completely sure of this. Strangely enough, the author was not able to create a consistent picture. The main problem was to understand contextuality’s physical meaning. The intensive discussions with the experimenters at the Atom Institute in Vienna did not help much. What specialties of quantum systems and measurement devices are reflected in the experimental violations [131,132] of non-contextual inequalities?

### 2.3. Jump from Contextuality to Bell Inequalities

Typically, by writing a paper about contextuality in QM, one starts by referring to this notion as *joint measurement contextuality* (JMC): “*dependence of the outcomes of some observable a on its joint measurement with another observable b* ”. I stress that this definition is countefactual and cannot be used in the experimental framework.

Nevertheless, the “universal contextuality writer” is not disappointed by this situation, and he immediately jumps to the Bell inequalities, which are treated as *noncontextual inequalities* (see, e.g., [41]). Moreover, contextuality is often identified with the violation of the Bell inequalities and Bell contextuality in our terminology. This identification overshadows the problem of the physical meaning of contextuality. One jumps from the problem of understanding to the calculation of a numerical quantity, the degree of the violation of some Bell inequality. Such inequalities are numerous. Additionally, they can be tested in different experimental situations and generate the permanent flow of highly recognized papers.

The author suggested the following critical illustration for this strategy (contextuality = violation of the Bell inequalities) [133]. Consider the notion of a *random sequence.* The theory of randomness is the result of an intensive research (Mises, Church, Kolmogorov, Solomovov, Chatin and Martin-Löf; see, e.g., the first part of author’s book [134]). This theoretical basis led to elaboration of the variety of randomness tests which are used to check whether some sequence of outputs of physical or digital random generator is random. However, in fact, it is possible to check only pseudo-randomness. The universal test of randomness exists, but the proof of its existence is nonconstructive, and this test cannot be applied to a concrete sequence of outcomes.

In applications, the NIST test (a batch of tests for randomness) is the most widely used. Thus, in the theory of randomness we also use tests, but beyond them, there is the well developed theory of randomness. In particular, this leads to understanding that even if a sequence *x* passed the NIST test, this does not imply that it is random. In principle, there can be found another test such that *x* would not pass it. The latter would not be a surprise.

In contrast to the above illustration, in QM, contextuality is per definition the violation of some noncontextual (Bell) inequality (at least for some authors). Hence, the theoretical notion is identified with the Bell test—in fact, the batch of tests corresponding to different Bell inequalities. (The Bell test for classicality plays the role of the NIST test for randomness). This is really bad!—not only from a theoretical viewpoint, but even from a practical one. As was mentioned, by working with randomness, people understand well that even passing the NIST test does not guarantee randomness. In QM, passing the Bell test is by definition equivalent to contextuality. This is a wrong strategy which leads to skews in handling quantum contextuality.

The above discussion on the notions of contextuality vs. randomness can be completed by a short remark on randomness of quantum statistical data. This is a good place to inform the reader that in experiments, detected individual photons fail to pass the standard randomness test, exhibiting strong correlations [135] which can be understood by analyzing the detectors properties [136] (see also [137] for the theoretical background of this analysis).

### 2.4. Signaling and Other Anomalies in Data

The first signs that addiction to one concrete test of contextuality (Bell inequalities) may lead to the wrong conclusions were observed by Adenier and Khrennikov [66,67,68,69,70,71]. Adenier was working on the translation of the PhD thesis of Alain Aspect (due to the joint agreement with Aspect and Springer), and he pointed out to me that he found some strange anomalies in Aspect’s data [72]. One of them was signaling. i.e., dependence of detection probability on one side (Bob’s laboratory) on the selection of an experimental setting on another side (Alice’s laboratory).

Then, we found signaling in the data from the famous Weihs experiment, closing the nonlocality loophole [73]. Our publications [66,67,68,69,70,71] attracted attention to the problem of signaling in data collected in quantum experiments. Slowly, people started to understand that the experimenter cannot be happy by just getting higher degree of the violation of, say, the CHSH inequality, with higher confidence. Often, this implies the increase in the degree of signaling. Experimenters started to check the hypothesis of signaling in data [117,119]. Unfortunately, our message was ignored by some experimenters; e.g., the data from the “the first loophole free experiment” [75] demonstrated statistically significant signaling.

*ny Bell test should be combined with the test of experimental statistical data on signaling.*

I pointed out that signaling was not the only problem in Aspect’s data. As he noted in his thesis [72], the data contain “anomalies” of the following type. Although the CHSH combination of correlations violates the CHSH inequality, the correlation for the concrete pair of angles $\theta_{1},\;\phi ,$ as a function of these angles does not match the theoretical prediction of QM. The graph of the experimental data differs essentially from the theoretical cos-graph. Our attempts to discuss this problem with other experimenters generated only replies that “we do not have such anomalies in our data”. (One of the problems in testing the Bell inequalities is the absence of openly approachable experimental data. I struggled a lot to create the open database. However, in spite of the positive responses, it seems that it was not created. The author of this paper even wrote an article, “Unuploaded experiments have no result” [138].)

### 2.5. Växjö Model for Contextual Probability

In the probabilistic terms of complementarity, the incompatibility of observables means that their *joint probability distribution* (JPD) does not exist. Instead of the JPD, one has to operate with a context-dependent family of probability spaces—*the Växjö contextual probability model* [25,26,27,28,29,30,31,32,33,34,35,36]:

$$M_{Z}=\left(P_{C},C\in Z\right),$$

where Z is a family of contexts, and for each C∈Z,

$$P_{C}=\left(\Omega_{C},F_{C},P_{C}\right)$$

is Kolmogorov probability space (Appendix B). Here, $\Omega_{C}$ is a sample space, $F_{C}$ is a σ-algebra of subsets of $\Omega_{C}$ (events) and $P_{C}$ is a probability measure on $F_{C}.$ All these structures depend on context C. To develop a fruitful theory, Z must satisfy some condition giving the possibility to create an analog of the CP calculus of conditional probabilities (see below).

In CP, the points of $\Omega_{C}$ represent elementary events, the most simple of which can happen within context C. Although these events are elementary, their structure can be complex and include the events corresponding to appearance of some parameters (“hidden variables”) for a system under observation and measurement devices, times of detection and so on.

Observables are given by random variables on contextually-labeled probability spaces, measurable functions and $a_{C}:\Omega_{C}\to R.$ The same semantically defined observable *a* is represented by a family of random variables $(a_{C},C\in Z_{a}),$ where $Z_{a}$ is the family of contexts for which the *a*-observable can be measured. In $M_{Z}$ averages are also labeled by contexts, $P_{a|C}$:

(1) $${\langle a\rangle }_{C}=E\left[a_{C}|P_{C}\right]=\int_{\Omega_{C}}a_{C}\left(\omega \right)dP_{C}\left(\omega \right)=\int_{R}xdp_{a}^{C}\left(x\right),$$

where the contextual probability distribution of the observable *a* is given by the equality

(2) $$p_{a}^{C}\left(x\right)\equiv P\left(a=x|C\right)=P\left(\omega \in \Omega_{C}:a_{C}\left(\omega \right)=x\right).$$

For contextual correlations, we have

(3) $${\langle ab\rangle }_{C}=E\left[a_{C}b_{C}|P_{C}\right]=\int_{\Omega_{C}}a_{C}\left(\omega \right)b_{C}\left(\omega \right)dP_{C}\left(\omega \right)=\int_{R^{2}}xydp_{a,b}^{C}\left(x,y\right),$$

where $p_{a,b}^{C}$ is the JPD of the pair of random variables $(a_{C},b_{C}).$ Here, *C* belongs to $Z_{a,b},$ the family of contexts for the joint measurement of observables *a* and b.

I remark that quantum logical-approach studies generalized probabilistic theories which are contextual in the sense described in this article. In this way, the Växjö model is a particular case of the general probabilistic framework based in measures over bounded lattices [139]. However, as such, the description of quantum probabilities is more specific, because it is described in terms of orthomodular lattices. In fact, the Växjö model is a step towards specialization of generalized probabilistic theories to match quantum theory. As was noted above, the collection of contexts Z satisfies some condition, giving the possibility to create an analog of the CP calculus of conditional probabilities. It is assumed that each observable *a* with the range of values $X_{a},\;$Z contains all contexts corresponding to the concrete outcomes of *a*-measurements and contexts $C_{x}^{a}.$ In probabilistic terms, they are characterized by the equality:

(4) $$P(a=x|C_{x}^{a})=1.$$

This equality is the purely probabilistic formalization of the projection postulate for operators with non-degenerate spectra. By operating with such contexts, we can introduce the transition probabilities

$$p\left(b=y|a=x\right)=P\left(b=y|C_{x}^{a}\right)$$

and derive the general contextual formula of total probability—the CP-formula is perturbed by the interference term [32,34,36,37,38]. In quantum theory, this calculus of transition probabilities is associated with *a*-observables given by Hermitian operators with non-degenerate spectra; $C_{x}^{a}$ corresponds to the *x*-eigenvector of the Hermitian operator and *A* represents the observable a.

In the series of papers [32,34,36,37,38], there was formulated and studied Born’s inverse problem—to reconstruct the complex Hilbert space representation of quantum probabilities from the Växjö model by operating with transition probabilities p(b=y|a=x). This problem is very complex, and it was solved only in some simple cases [37,38].

However, this part of the Växjö model (the calculus of transition probabilities) is not relevant to the joint measurements of observables. It is useful for probabilistic modeling of the sequential measurements. Thus, it is irrelevant to the main theme of this article.

### 2.6. Summary of the Preliminary Discussion

The discussion can be concluded with a few statements:

- The theoretical definition of contextuality as JMC suffers from appealing to conterfactuals.
- Identification of contextuality with the violation of the Bell inequalities is not justified, either physically or mathematically (in the last case, such an approach does not match the mathematical tradition).
- The Bell tests should be accompanied with tests of signaling.
- “Unuploaded to internet experiments have no results” [138].
- Probabilistically, contextuality–complementarity is described by contextual probability (as by the Växjö model).

## 3. Thinking over Bohr’s Ideas

This section is devoted to thinking over Bohr’s foundational works in terms of contextuality.

### 3.1. Bohr Contextuality

The crucial question is about the physical meaning of contextuality; without answering this issue, JMC (even by ignoring counterfactuality) is mystical, especially for spatially separated systems. Even spooky action at a distance is welcome—to resolve this mystery.

In the series of papers [5,6,7,8,9,10,11,12,13,14,15,16], the physical meaning of contextuality was clarified through referring to Bohr’s complementarity principle. Typically, this principle is reduced to wave–particle duality. (In fact, Bohr had never used the latter terminology). However, Bohr’s formulation of the complementarity principle is essentially deeper. Complementarity is not postulated; for Bohr, it is the natural consequence of the irreducible dependence of observable’s outcome on the experimental context. Thus, the outcomes of quantum observables are generated in the complex process of the interaction of a system and a measurement device [4] (see also [15,140]). This dependence on the complex of experimental conditions is nothing other than a form of contextuality, *Bohr contextuality* (Section 3.2). I remark that JMC is its special case. However, in contrast to JMC, the physical interpretation of Bohr contextuality is transparent dependence of results of measurements on experimental contexts. Additionally, it does not involve the use of conterfactuals.

Such contextuality is the seed of complementarity, the existence of incompatible observables. (Recall that observables are incompatible if they cannot be measured jointly). Moreover, contextuality without incompatibility loses its value.

*If all observables were compatible, then they might be jointly measured in a single experimental context, and multicontextual consideration would be meaningless.*

One can go in deeper foundations of QM and ask:

*Why is dependence on experimental context (system–apparatus interaction) irreducible?*

Bohr’s answer is that irreducibility is due to the existence of the *indivisible quantum of action* given by the Planck constant (see articles [8,9] for discussion and references).

I stress that the interactions considered in quantum theory are not the classical force-like interactions. QM interactions are represented by unitary operators, and they follow the “physical laws” of quantum theory. This is the operational viewpoint on interaction which is widely used in theory of open quantum system. It is mathematically formalized in the indirect measurement scheme and the mathematical construction of quantum instruments from unitary interaction between a system and apparatus [10,11,12,13,14]. (See article [15] for a formalization of Bohr’s contextuality within the indirect measurement scheme.)

### 3.2. Bohr’s Principle of Contextuality–Complementarity

The Bohr principle of complementarity [4] is typically presented as wave–particle duality, incompatibility of the position and momentum observables. The latter means the impossibility of their joint measurement. I remark that Bohr started with the problem of incompatibility of these observables by discussing the two slit experiment. In this experiment, position is represented by the “which slit?” observable, and momentum is determined by the detection dot on the registration screen. (This screen is covered by photo-emulsion and placed at some distance beyond the screen with two slits.) Later, Bohr extended the wave–particle duality to arbitrary observables, which cannot be jointly measured, and formulated the principle of complementarity. He justified this principle by emphasizing the contextuality of quantum measurements. Bohr’s viewpoint on contextuality was wider than in the modern discussion on quantum contextuality related to the Bell inequality. The latter is the contextuality of joint measurement with a compatible observable (Section 2.3).

In 1949, Bohr [4] presented the essence of complementarity in the following widely cited statement:

“*This crucial point … implies the impossibility of any sharp separation between the behaviour of atomic objects and the interaction with the measuring instruments which serve to define the conditions under which the phenomena appear. In fact, the individuality of the typical quantum effects finds its proper expression in the circumstance that any attempt of subdividing the phenomena will demand a change in the experimental arrangement introducing new possibilities of interaction between objects and measuring instruments which in principle cannot be controlled. Consequently, evidence obtained under different experimental conditions cannot be comprehended within a single picture, but must be regarded as complementary in the sense that only the totality of the phenomena exhausts the possible information about the objects*”.

In short, Bohr’s way to the complementarity principle, the claim on the existence of incompatible quantum observables, can be presented as the following chain of reasoning [5,6,7,8,9,10,11,12,13,14,15,16]:

- **CONT1:** An outcome of any observable is composed of the contributions of a system and a measurement device. (Thus, the values of an observable *a* are not the objective properties of the systems. They are created in the process of the complex interaction between the systems prepared for measurements and the apparatus used for measurement of a.)
- **CONT2**: The whole experimental context has to be taken into account.
- **INCOMP1**: There is no reason to expect that all experimental contexts can be combined with each other and all observables can be measured jointly; thus, some observables can be incompatible.
- **INCOMP2**: The Heisenberg uncertainty principle implies that the position and momentum observables are incompatible.

The statements **CONT1** + **CONT2** and **INCOMP1** + **INCOMP12** compose the contextual and incompatibility parts of Bohr’s reasoning. Bohr considered the two-slit experiment as the experimental confirmation of the Heisenberg uncertainty principle. Hence, for him, **INCOMP2** was experimentally verified.

In the light of such structuring of Bohr’s thinking, it might be more natural to speak about two Bohr’s principles:

- *Contextuality Principle.*
- *Complementarity Principle*.

We can unify these two principles and speak about the **contextuality–complementarity principle**, instead of simply the complementarity principle. Unfortunately, the contextual dimension of the complementarity is typically missing in the discussions on quantum foundations.

Of course, the fact that outcomes of observables depend irreducibly on experimental conditions does not imply the existence of other experimental conditions which are incompatible. However, if the outcomes were the objective properties of physical systems, then at least in principle any two experimental contexts would be combinable.

By speaking that the wave and particle properties cannot be merged in a single experimental framework (the wave–particle duality), we have to remember that the seed of their dispersing is contextuality: they are determined within two different experimental contexts.

This is a good place to remark that it is possible to establish wave–particle correlations in the conditional experiments, as in article [141], where amplitude of the electromagnetic field was conditioned to photon detection. This experiment supports the treatment of quantum probabilities as conditional ones.

In the light of Bohr-contextuality, the following natural question arises:

*How can one prove that the concrete observables a and b cannot be jointly measured (i.e., that they are incompatible)?*

From the viewpoint of experimental verification, the notion of incompatibility is difficult. How can one show that the joint measurement of *a* and *b* is impossible? One can refer to the mathematical formalism of quantum theory and say that the observables *a* and *b* cannot be jointly measurable if the corresponding Hermitian operators *A* and *B* do not commute. However, another debater can say that may be this is just the artifact of the quantum formalism: yes, the operators do not commute, but observables still can be jointly measured. The latter argument was used by some experts in quantum foundations against the appeal to the Heisenberg uncertainty principle as justification of the existence of incompatible observables—**INCOMP2** (see Appendix A).

## 4. Probabilistic Viewpoint on Contextuality–Complementarity

The basic analysis on the (in)compatibility problem is done in probabilistic terms. Suppose that observables a,b,c,… can be in principle jointly measured, but we are not able to design the corresponding measurement procedure. Nevertheless, the assumption of joint measurability, even hypothetical, implies the existence of JPD.

*What are consequences of JPD’s existence?*

We shall come back to this question in Section 4.1. Now, I remark that the principle of contextuality–complementarity can be reformulated in probabilistic terms. In short, we can say that *the measurement part of QM is a (special) calculus of context-dependent probabilities.* This viewpoint was presented in a series of works summarized in monograph [34] devoted to the calculus of context-dependent probability measures $(P_{C}),C\in Z,$ where Z is a family of contexts constrained by some consistency conditions.

I emphasize that QP is a special contextual probabilistic calculus. *Its specialty consists of the possibility to use a quantum state (the wave function) |ψ〉 to unify generally incompatible contexts.* This is the important feature of QP playing the crucial role in quantum foundations.

In classical statistical physics, the contextuality of observations is not emphasized. Here it is assumed that it is possible to proceed in the CP framework to introduce a single context-independent probability measure *P* and reproduce the probability distributions of all physical observables on the basis of P. This is really possible. However, the careful analysis of interplay of probability measures appearing in classical physics shows that even here, contexuality cannot be ignored. In articles [142,143], there are considered models, e.g., in the theory of complex disordered systems (spin glasses), such that it is impossible to operate with just one fixed probability measure P. A variety of context dependent probabilities have to be explored. I especially emphasize the paper on classical probabilistic entanglement [144].

### 4.1. Existence vs. Non-Existence of the Joint Probability Distribution

Let P=(Ω,F,P) be a Kolmogorov probability space [145]. Each random variable a:Ω→R determines the probability distribution $P_{a}.$ The crucial point is that all these distributions are encoded in the same probability measure $P:P_{a}\left(\alpha \right)=P\left(\omega \in \Omega :a\left(\omega \right)=\alpha \right).$ (I consider only discrete random variables.)

*In CP, the probability distributions of all observables (represented by random variables) can be consistently unified on the basis of P.*

For any pair of random variables *a* and b, their JPD $P_{a,b}$ is defined, and the following condition of *marginal consistency* holds:

(5) $$P_{a}\left(\alpha \right)=\sum_{\beta }P_{a,b}\left(\alpha ,\beta \right)$$

This condition means that observation of *a* jointly with *b* does not change the probability distribution of a. Equality (5) implies that, for any two observables *b* and c,

(6) $$\sum_{\beta }P_{a,b}\left(\alpha ,\beta \right)=\sum_{\gamma }P_{a,c}\left(\alpha ,\gamma \right).$$

In fact, condition (6) is equivalent to (5): by selecting the random variable *c* such that c(ω)=1 almost everywhere, we see that (6) implies (5). These considerations are easily generalized to a system of *k* random variables $a_{1},\ldots ,a_{k}.$ Their JPD is well defined:

$$P_{a_{1},\ldots ,a_{k}}\left(\alpha_{1},\ldots ,\alpha_{k}\right)=P\left(\omega \in \Omega :a_{1}\left(\omega \right)=\alpha_{1},\ldots ,a_{k}\left(\omega \right)=\alpha_{k}\right).$$

Additionally, marginal consistency conditions hold for all subsets of random variables $(a_{i_{1}},\ldots ,a_{i_{m}}),\;$m<k).

Consider now some system of *experimental observables* $a_{1},\ldots ,a_{k}.$ If the experimental design for their joint measurement exists, then it is possible to define their JPD $P_{a_{1},\ldots ,a_{k}}\left(\alpha_{1},\ldots ,\alpha_{k}\right)$ (as the relative frequency of their joint outcomes). This probability measure $P\equiv P_{a_{1},\ldots ,a_{k}}$ can be used to define the Kolmogorov probability space; i.e., the case of joint measurement can be described by CP.

Now consider the general situation: only some groups of observables can be jointly measured. For example, there are three observables, a,b and *c*, and only the pairs (a,b) and (a,c) can be measurable; i.e., only JPDs $P_{a,b}$ and $P_{a,c}$ can be defined and associated with the experimental data. There is no reason to assume the existence of JPD $P_{a,b,c}.$ In this situation equality, (6) may be violated. In the terminology of QM, this violation is called *signaling.*

Typically, one considers two laboratories, Alice’s and Bob’s laboratories. Alice measures the *a*-observable, and Bob can choose whether to measure the *b*- or *c*-observable. If

(7) $$\sum_{\beta }P_{a,b}\left(\alpha ,\beta \right)\neq \sum_{\gamma }P_{a,c}\left(\alpha ,\gamma \right),$$

one says that the *a*-measurement procedure is disturbed (in some typically unknown way) by the selection of a measurement procedure by Bob. Some signal from Bob’s laboratory approaches Alice’s laboratory and changes the probability distribution. This terminology, signaling vs. no-signaling, is adapted to measurements on spatially separated systems and related to the issue of nonlocality. In quantum-like models, one typically works with spatially localized systems and is interested in contextuality (what ever it means). Therefore, I called condition (6) marginal consistency (consistency of marginal probabilities), and (7) is marginal inconsistency. In the remainder of the text, we shall use both terminologies interchangeably—marginal consistency vs. inconsistency and no-signaling vs. signaling.

We should now be mainly interested in the CHSH inequality. In this framework, we shall work with four observables, $a_{1},a_{2}$ and $b_{1},b_{2};$ experimenters are able to design measurement procedures only for some pairs of them, say, $(a_{i},b_{j}),i,j=1,2.$ In this situation, there is no reason to expect that one can define (even mathematically) the JPD $P_{a_{1},a_{2},b_{1},b_{2}}\left(\alpha_{1},\alpha_{2},\beta_{1},\beta_{2}\right).$ This situation is typical for QM. This is a complex interplay of theory and experiment. Only probability distributions $P_{a_{i},b_{j}}$ can be experimentally verified. However, in theoretical speculation, we can consider JPD $P_{a_{1},a_{2},b_{1},b_{2}}$ as a *mathematical quantity.* If it existed, we might expect that there would be some experimental design for joint measurement of the quadruple of observables $(a_{1},a_{2},b_{1},b_{2}).$ On the other hand, if it does not exist, then it is meaningless even to try to design an experiment for their joint measurement.

Now we turn back to marginal consistency: in general (if $P_{a_{1},a_{2},b_{1},b_{2}}$ does not exist), it may be violated. However, in QM it is not violated: *there is no signaling.* This is the miracle feature of QM. Often it is coupled to spatial separation of systems: $a_{1}$ or $a_{2}$ are measured on $S_{1}$ and $b_{1}$ or $b_{2}$ on $S_{2}.$ Additionally, these systems are so far from each other that the light signal emitted from Bob’s laboratory cannot approach Alice’s laboratory during the time of the measurement and manipulation with the selection of experimental settings. However, as we shall see, no-signaling is the general feature of the quantum formalism, which has nothing to do with spatial separability, nor even with consideration of the compound systems.

## 5. Clauser, Horne, Shimony and Holt (CHSH) Inequality

I restrict further considerations to the CHSH framework; i.e., we shall not consider other types of Bell inequalities.

How can one get to know whether JPD exists? The answer to this question is given by a theorem of Fine [40] concerning the CHSH inequality.

Consider dichotomous observables $a_{i}$ and $b_{j}\left(i,j=1,2\right)$, taking values ±1. In each pair $\left(a_{i},b_{j}\right)$, observables are compatible, i.e., they can be jointly measurable, and pairwise JPDs $P_{a_{i},b_{j}}$ are well defined. Consider correlation

$$\langle a_{i}b_{j}\rangle =E\left[a_{i}b_{j}\right]=\int \alpha \beta \;dP_{a_{i},b_{j}}\left(\alpha ,\beta \right);$$

in the discrete case,

$$\langle a_{i}b_{j}\rangle =E\left[a_{i}b_{j}\right]=\sum_{\alpha \beta }\alpha \beta \;P_{a_{i},b_{j}}\left(\alpha ,\beta \right).$$

By Fine’s theorem, JPD $P_{a_{1},a_{2},b_{1},b_{2}}$ exists if and only if the CHSH inequality for these correlations is satisfied:

(8) $$|\langle a_{1}b_{1}\rangle +\langle a_{1}b_{2}\rangle +\langle a_{2}b_{1}\rangle -\langle a_{2}b_{2}\rangle |\leq 2.$$

and the three other inequalities correspond to all possible permutations of indexes i,j=1,2.

### 5.1. Derivation of CHSH Inequality within Kolmogorov Theory

The crucial assumption for derivation of the CHSH inequality is that all correlations are with respect to the same Kolmogorov probability space P=(Ω,F,P) and that all observables $a_{i},b_{j},i,j=1,2,$ can be mathematically represented as random variables on this space. Under the assumption of the JPD existence, one can select the sample space $\Omega ={\left\{-1,+1\right\}}^{4}$ and the probability measure $P=P_{a_{1},a_{2},b_{1},b_{2}}.$ Thus, the CHSH inequality has the form

(9) $$|\int_{\Omega }\left[a_{1}\left(\omega \right)b_{1}\left(\omega \right)+a_{1}\left(\omega \right)b_{2}\left(\omega \right)+a_{2}\left(\omega \right)b_{1}\left(\omega \right)-a_{2}\left(\omega \right)b_{2}\left(\omega \right)\right]dP\left(\omega \right)|\leq 2.$$

The variable ω can include hidden variables of a system, measurement devices, detection times and so on. Only important is the possibility to use the same probability space to model all correlations. The latter is equivalent to the existence of JPD $P_{a_{1},a_{2},b_{1},b_{2}}.$ This is the trivial part of Fine’s theorem; JPD implies the CHSH inequality. The other way around is more difficult [40].

This inequality can be proven by integration of the inequality

$$-2\leq a_{1}\left(\omega \right)b_{1}\left(\omega \right)+a_{1}\left(\omega \right)b_{2}\left(\omega \right)+a_{2}\left(\omega \right)b_{1}\left(\omega \right)-a_{2}\left(\omega \right)b_{2}\left(\omega \right)\leq 2$$

which is the consequence of the inequality

$$-2\leq a_{1}b_{1}+a_{1}b_{2}+a_{2}b_{1}-a_{2}b_{2}\leq 2$$

which holds for any quadrupole of real numbers belonging to [−1, +1].

### 5.2. Role of No-Signaling in Fine’s Theorem

The above presentation of Fine’s result is common for physics’ folklore. However, Fine did not consider explicitly the CHSH inequalities presented above; see (8). He introduced four inequalities that are necessary and sufficient for the JPD to exist, but these inequalities are expressed differently to the CHSH inequalities. The CHSH inequalities are derivable from Fine’s four inequalities stated in Theorem 3 of his paper.

I remark that the existence of the quadruple JPD implies marginal consistency (no-signaling). Additionally, Fine’s theorem presupposed that marginal consistency.

This is a good place to make the following remark. In quantum physics, this very clear and simple meaning of violation of the CHSH inequality (non-existence of JPD) is obscured by the issue of nonlocality. However, in this article, I am not aiming to criticize the nonlocal interpretation of QM. If somebody speaks about spooky action at a distance and other mysteries of QM, I have no quarrel with this, since I only use the quantum formalism, not its special interpretation. Finally, I point out that the Bell-type inequalities were considered already by Boole (1862) [146,147] as necessary conditions for the existence of a JPD.

### 5.3. Violation of CHSH Inequality for Växjö Model

If it is impossible to proceed with the same probability space for all correlations, one has to use the Växjö model (Section 2.5), and there is no reason to expect that the following inequality (and the corresponding permutations) would hold:

(10) $$|\int_{\Omega_{C_{11}}}a_{C_{11}}\left(\omega \right)b_{C_{11}}\left(\omega \right)dP_{C_{11}}\left(\omega \right)+\int_{\Omega_{C_{12}}}a_{C_{12}}\left(\omega \right)b_{C_{12}}\left(\omega \right)dP_{C_{12}}\left(\omega \right)+$$

$$\int_{\Omega_{C_{21}}}a_{C_{21}}\left(\omega \right)b_{C_{21}}\left(\omega \right)dP_{C_{21}}\left(\omega \right)-\int_{\Omega_{C_{22}}}a_{C_{22}}\left(\omega \right)b_{C_{22}}\left(\omega \right)dP_{C_{22}}\left(\omega \right)|\leq 2,$$

where $C_{ij}$ is the context for the joint measurement of the observables $a_{i}$ and $b_{j}.$ Here, $a_{i}$-observable is represented by random variables $(a_{C_{i1}}$ and $a_{C_{i2}})$, and $b_{i}$-observable by random variables $(b_{C_{1i}}$ and $b_{C_{2i}}).$

In the Växjö model the condition of no-signaling may be violated; for discrete variables, signaling means that

$$\sum_{y}P_{a_{1},b_{1}}^{C_{11}}\left(x,y\right)\neq \sum_{y}P_{a_{1},b_{2}}^{C_{12}}\left(x,y\right).$$

## 6. CHSH Inequality for Quantum Observables: Representation via Commutators

In this section, I present the purely quantum treatment of the CHSH inequality and highlight the role of incompatibility in its violation (I follow article [6]). Although in QM the CHSH inequality is typically studied for compound systems with the emphasis on the tensor product structure of the state space, in this section I shall not emphasize the latter and proceed for an arbitrary state space and operators. Consequences and simplifications for the tensor product case will be presented in Section 6.1.

Observables $a_{i},b_{j}$ are described by (Hermitian) operators $A_{i},B_{j},i,j=1,2,$

(11) $$[A_{i},B_{j}]=0,i,j=1,2.$$

I remark that generally

$$\left[A_{1},A_{2}\right]\neq 0,\;\left[B_{1},B_{2}\right]\neq 0;$$

i.e., the observables in the pairs $a_{1},a_{2}$ and $b_{1},b_{2}$ do not need to be compatible.

Observables under consideration are dichotomous with values ±1. Hence, the corresponding operators are such that $A_{i}^{2}=B_{j}^{2}=I.$ The latter plays the crucial role in derivation of the Landau equality (15).

Consider the CHSH correlation represented in the quantum formalism and normalized by 1/2:

(12) $$\langle B\rangle =\frac{1}{2}\left[\langle A_{1}B_{1}\rangle +\langle A_{1}B_{2}\rangle +\langle A_{2}B_{1}\rangle -\langle A_{2}B_{2}\rangle \right].$$

This correlation is expressed via the Bell operator:

(13) $$B=\frac{1}{2}\left[A_{1}\left(B_{1}+B_{2}\right)+A_{2}\left(B_{1}-B_{2}\right)\right]$$

as

(14) 〈B〉=〈ψ|B|ψ〉.

Simple calculations lead to the Landau identity [148,149]:

(15) $$B^{2}=I-\left(1/4\right)\left[A_{1},A_{2}\right]\left[B_{1},B_{2}\right].$$

If *at least one commutator* equals to zero, i.e.,

(16) $$[A_{1},A_{2}]=0,$$

or

(17) $$[B_{1},B_{2}]=0,$$

then, for quantum observables, we obtain the inequality

(18) |〈B〉|≤1.

Derivation of (18) was based solely on quantum theory. This inequality is the consequence of compatibility for at least one pair of observables, $A_{1},A_{2}$ or $B_{1},B_{2}.$ Symbolically, Equation (18) is the usual CHSH inequality, but its meaning is different. Equation (18) can be called *the quantum CHSH inequality.*

Now suppose that $A_{i}$-observables and $B_{j}$-observables are incompatible—i.e., the corresponding operators do not commute:

(19) $$\left[A_{1},A_{2}\right]\neq 0\;and\;\left[B_{1},B_{2}\right]\neq 0,$$

i.e.,

(20) $$M_{A}\neq 0\;and\;M_{B}\neq 0,$$

where the commutator observables are defined as $M_{A}=i\left[A_{1},A_{2}\right],\;M_{B}=i\left[B_{1},B_{2}\right].$ I emphasize that

$$[M_{A},M_{B}]=0.$$

The Landau identity can be written as

(21) $$B^{2}=I+\left(1/4\right)M_{AB},$$

where $M_{AB}=M_{A}M_{B}=M_{B}M_{A}$ is the operator of the composition of commutator operators.

I remark that if $M_{AB}=0,$ then, in spite the incompatibility condition (19), the quantum QCHSH inequality cannot be violated. Thus, we can continue under the condition that

(22) $$M_{AB}\neq 0.$$

This condition is not so restrictive. In the author’s interpretation, the quantum CHSH inequality is simply one of possible statistical tests of incompatibility. It provides the possibility to estimate the degree of incompatibility in a pair of observables, e.g., in the *A* pair. The *B* pair is the auxiliary; it can be selected by experimenters.

The condition in Equation (22) is guaranteed via selection of the *B*-operators in such a way that the operator $M_{B}$ is invertible. I point out that the case of compound systems (see Section 6.1) with incompatibility of the *A*-observables and the *B*-observables implies the non-degeneration condition (22).

Under condition (22), there exists common eigenvector $\psi_{AB}$ of commuting commutator operators,

$$M_{A}\psi_{AB}=\mu_{A}\psi_{AB},M_{B}\psi_{AB}=\mu_{B}\psi_{AB},$$

such that both eigenvalues $\mu_{A},\mu_{B}$ are nonzero.

Consider the case when $\mu_{A}>0$ and $\mu_{B}>0.$ Such $\psi_{AB}$ is an eigenvector of operator $B^{2}$ with eigenvalue $\left(1+\mu \right)>1,\mu =\mu_{A}\mu_{B}.$ Thus, $∥B^{2}∥\;\geq \;\left(1+\mu \right)>1$ and

$$1<\left(1+\mu \right)\;\leq \;∥B^{2}{∥\;=\;∥B∥}^{2}.$$

Operator B is a Hermitian, and this implies that

$$∥B∥=\underset{∥\psi ∥=1}{\sup}|\langle \psi |B|\psi \rangle |.$$

Finally, we obtain the following estimate:

$$\underset{∥\psi ∥=1}{\sup}|\langle \psi |B|\psi \rangle |>\sqrt{1+\mu }>1.$$

I demonstrated that, for some pure states, the quantum CHSH inequality f is violated.

Consider now the case $\mu_{A}>0,$ but $\mu_{B}<0.$ The sign of $\mu_{B}$ can be changed via interchange the *B*-observables.

I conclude:

*Conjunction of incompatibilities of the A-observables and the B-observables constrained by Equation (22) is sufficient for violation of the quantum CHSH inequality (for some quantum state).*

The degree of violation can serve as an incompatibility measure in two pairs of quantum observables, $A_{1},A_{2}$ and $B_{1},B_{2}.$ Testing the degree of incompatibility is testing the degree of noncommutativity, or in other words, the ”magnitudes” of observables corresponding to commutators

(23) $$M_{A}=i\left[A_{1},A_{2}\right],\;M_{B}=i\left[B_{1},B_{2}\right].$$

The incompatibility magnitude can be expressed via the maximal value of averages of commutator operators, i.e., by their norms; for example,

(24) $$\underset{∥\psi ∥=1}{\sup}\left|\langle \psi \right|M_{A}|\psi \rangle |\;=\;∥M_{A}∥.$$

By interpreting quantity $\langle \psi |M_{A}|\psi \rangle$ as the theoretical counterpart of experimental average ${\langle M_{A}\rangle }_{\psi }$ of observable $M_{A},$ we can measure experimentally the incompatibility magnitude, i.e., norm $∥M_{A}∥$ from measurements of commutator-observable $M_{A}.$ (The main foundational problem is that measurement of such commutator observables is challenging. Recently, some progress was demonstrated on the basis of weak measurements, but generally we are not able to measure commutator quantities.)

I remark that (from the quantum mechanical viewpoint) the CHSH test estimates the product of incompatibility magnitudes for the *A*-observables and *B*-observables, i.e., the quantity $∥M_{A}∥∥M_{B}∥.$ By considering the *B*-observables as auxiliary and selecting them in a proper way (for example, such that the *B*-commutator is a simple operator), we can use the CHSH test to obtain the experimental value for the incompatibility magnitude given by $∥M_{A}∥.$

### 6.1. Compound Systems: Incompatibility as a Necessary and Sufficient Condition of Violation of a Quantum CHSH Inequality

Here, $H=H_{A}⊗H_{B}$ and $A_{j}=A_{j}⊗I,B_{j}=I⊗B_{j},$ where Hermitian operators $A_{j}$ and $B_{j}$ act in $H_{A}$ and $H_{B}$, respectively.

Here, the joint incompatibility-condition in Equation (19) is equivalent to incompatibility of observables on subsystems:

(25) $$M_{A}=i\left[A_{1},A_{2}\right]\neq 0\;and\;M_{B}=i\left[B_{1},B_{2}\right]\neq 0.$$

We have $M_{AB}=M_{A}M_{B}=M_{A}⊗M_{B}.$ As mentioned above, constraint $M_{AB}\neq 0$ is equivalent to (25). Thus, *conjunction of local incompatibilities* is the sufficient condition for violation of the quantum CHSH inequality. Additionally, we obtain:

**Theorem** **1** (Local incompatibility criteria of CHSH violation). *Conjunction of local incompatibilities is the necessary and sufficient condition for violation of the quantum CHSH inequality.*

### 6.2. Tsirelson Bound

By using Landau identity (15) we can derive the *Tsirelson bound* $2\sqrt{2}$ for the CHSH correlation of quantum observables, i.e., observables which are represented by Hermitian operators $A_{i},B_{j},i,j=1,2,$ with spectrum ±1, so $A_{i}^{2}=B_{j}^{2}=I.$ For such operators, for any state |ψ〉, we have:

(26) $$|\langle A_{1}B_{1}\rangle +\langle A_{1}B_{2}\rangle +\langle A_{2}B_{1}\rangle -\langle A_{2}B_{2}\rangle |\leq 2\sqrt{2}.$$

On the other hand, if observables are not described by QM, then this bound can be exceeded. For the Växjö contextual probability model, the CHSH correlation may approach the value 4; the same is true for CbD model.

## 7. Signaling in Physical and Psychological Experiments

By using the quantum calculus of probabilities, it is easy to check whether the no-signaling condition holds for quantum observables, which are represented mathematically by Hermitian operators. Therefore, Fine’s theorem is applicable to quantum observables. This theoretical fact played an unfortunate role in hiding from view signaling in experimental research on the violation of the CHSH inequality. Experimenters were focused on observing as high a violation of (8) as possible, and they ignored the no-signaling condition. However, if the latter is violated, then a JPD automatically does not exist, and there is no reason to expect that (8) would be satisfied. The first paper in which the signaling issue in quantum experimental research was highlighted was Adenier and Khrennikov (2006) [66]. There it was shown that statistical data collected in the basic experiments (for that time) performed by Aspect [72] and Weihs [73] violate the no-signaling condition.

After this publication, experimenters became aware of the signaling issue and started to check it [117,119]. However, analysis presented in Adenier and Khrennikov [71] demonstrated that even statistical data generated in the first loophole-free experiment to violate the CHSH inequality [75] exhibit very strong signaling. Nowadays, the no-signaling condition is widely discussed in quantum information theory, but without referring to the pioneering works of Adenier and Khrennikov [66,67,68,69,70,71].

The experiments to check CHSH and other Bell-type inequalities were also performed for mental observables in the form of questions asked to people [106,109,110,111,112,113,114,115,116]. The first such experiment was done in 2008 [109] and was based on the theoretical paper of Khrennikov [150]. As was pointed out by Dzhafarov et al. [113], all known experiments of this type suffer from signaling. Moreover, in contrast to physics, in psychology there are no theoretical reasons to expect no-signaling. In this situation, Fine’s theorem is not applicable. Additionally, Dzhafarov and his coauthors were the first who understood the need for adapting the Bell-type inequalities to experimental data exhibiting signaling. Obviously, the interplay of whether or not a JPD exists for the quadruple of observables

(27) $$s=(a_{1},a_{2},b_{1},b_{2})$$

cannot be considered for signaling data.

## 8. Contextuality by Default

### 8.1. Contextual Indexing

Dzhafarov and his coauthors [78,79,80,81,82,83] proposed to consider, instead of a quadruple of observables s, the corresponding octuple s, which is generated by doubling each observable and associating s with four contexts of measurements of pairs,

(28) $$C_{11}=\left(a_{1},b_{1}\right),C_{12}=\left(a_{1},b_{2}\right),C_{21}=\left(a_{2},b_{1}\right),C_{22}=\left(a_{2},b_{2}\right).$$

Thus, the basic object of CbD theory (for the CHSH inequality) is an octuple of observables

(29) $$s=\left(\left(a_{11},b_{11}\right),\left(a_{12},b_{21}\right),\left(a_{21},b_{12}\right),\left(a_{22},b_{22}\right)\right),$$

so, e.g., observable $a_{1}$, measured jointly with observable $b_{j}$, is denoted $a_{1j}.$

The essence of CbD is a coupling of observables to contexts. This coupling generates double-indexing of observables. The mathematical representations of the observable $a_{i}$ measured jointly with the observables $b_{j},j=1,2$ cannot be identified. This is the basic foundational counterpart of CbD.

CbD uses the random variables language to present observables, so it operates with four pairs of random variables:

(30) $$S=\left(\left(A_{11},B_{11}\right),\left(A_{12},B_{21}\right),\left(A_{21},B_{12}\right),\left(A_{22},B_{22}\right)\right),$$

and generally each pair of random variables is defined on its own probability space;

$$P_{ij}=\left(\Omega_{ij},F_{ij},P_{ij}\right).$$

Thus, CbD and the Växjö contextual probability model have the same foundational basis and operate with the families of Kolmogorov probability spaces corresponding to measurement contexts. However, technically they diverge.

### 8.2. Coupling Method

The main mathematical apparatus of CbD is the coupling method which is widely used in CP [86,87]. The Växjö model differs from CbD by the calculus of transition probabilities.

Let $P_{i}=\left(\Omega_{i},F_{i},P_{i}\right),i=1,2,$ be two Kolmogorov probability spaces, and let $X_{i}$ be random variables on these spaces. Then a coupling of $X_{1}$ and $X_{2}$ be a new probability space P=(Ω,F,P), over which there are two random variables $Y_{1}$ and $Y_{2}$ such that $Y_{1}$ has the same distribution as $X_{1}$ and $Y_{2}$ has the same distribution as $X_{2}.$

In the CHSH framework, the coupling method is applied as follows. Let $P_{ij}=\left(\Omega_{ij},F_{ij},P_{ij}\right)$ be probability spaces with vector random variables $(A_{ij},B_{ji});$ then their coupling is a probability space P=(Ω,F,P) over which there are defined four vector random variables $\left(A_{ij},B_{ji}\right)$, having the same probability distributions as the vectors $(A_{ij},B_{ji}).$

The coupling method can be considered as a mechanism of embedding of the contextual probability model into CP. This embedding gives the possibility to apply the mathematical apparatus of CP for generally non-Kolmogorovian model. Such an embedding is not unique, and in CbD one searches for an optimal (in some sense; see below) coupling. I remark that the Växjö model can also be embedded in some Kolmogorov probability space (see Section 9), and with such embedding the contextual probabilities are realized as the classical conditional probabilities.

Thus, the coupling technique gives the possibility to operate with a variety of octuples of random variables:

(31) $$S=(A_{11},B_{11},A_{12},B_{21},A_{21},B_{12},A_{22},B_{22}),$$

defined on the same probability space and giving couplings for the system of contextual random variables S.

### 8.3. The Problem of Identity, Its Resolution and Introduction of a Measure of Contextuality

By moving from quadruple *S* to octuple S, one confronts the problem of the identity of an observable which is now represented by two different random variables; e.g., the observable $a_{i}$ is represented by the random variables $A_{ij}\left(\omega \right),j=1,2.$ In the presence of signaling, one cannot expect the equality of two such random variables almost everywhere. Dzhafarov et al. came up with a novel treatment of the observable-identity problem.

It is assumed that averages

(32) $$m_{a;ij}=\langle A_{ij}\rangle ,\;m_{b;ij}=\langle B_{ij}\rangle$$

and covariation

(33) $$C_{ij}=\langle A_{ij}B_{ji}\rangle$$

are fixed. These are measurable quantities. They can be statistically verified by experiment.

Set

(34) $$\delta \left(a_{i}\right)=m_{a;i1}-m_{a;i2},\;\delta \left(b_{j}\right)=m_{b;j1}-m_{b;j2},$$

and

(35) $$\Delta_{0}=\frac{1}{2}\left(\sum_{i}\delta \left(a_{i}\right)+\sum_{j}\delta \left(b_{j}\right)\right).$$

This is the experimentally verifiable measure of signaling.

I remark that in the coupling representation, the joint satisfaction of the CHSH inequalities, i.e., (8) and other inequalities obtained from it via permutations, can be written in the form:

(36) $$\max_{ij}\left|\langle A_{11}B_{11}\rangle +\langle A_{12}B_{21}\rangle +\langle A_{21}B_{12}\rangle +\langle A_{21}B_{22}\rangle -2\langle A_{ij}B_{ji}\rangle \right|\leq 2.$$

In the signaling-free situation, e.g., in quantum physics, the difference between the left-hand and right-hand sides is considered as the measure of contextuality. Denote (1/2 times) this quantity by $\Delta_{\mathrm{CHSH}}.$ It is also experimentally verifiable.

Then, Dzhafarov and coauthors introduced quantity

(37) $$\Delta \left(P\right)=\sum \Delta_{a_{i}}\left(P\right)+\sum \Delta_{b_{j}}\left(P\right),$$

where

(38) $$\Delta_{a_{i}}\left(P\right)=P\left(\omega :A_{i1}\left(\omega \right)\neq A_{i2}\left(\omega \right)\right),\Delta_{b_{j}}\left(P\right)=P\left(\omega :B_{j1}\left(\omega \right)\neq B_{j2}\left(\omega \right)\right).$$

Here, $\Delta_{a_{i}}\left(P\right)$ characterizes mismatching of representations of observable $a_{i}$ by random variables $A_{i1}$ and $A_{i2}$ with respect to probability measure P; $\Delta_{b_{j}}\left(P\right)$ is interpreted in the same way. The problem of the identity of observables is formulated as the mismatching minimization or identity maximization problem

(39) Δ(P)→min

with respect to all octuple probability distributions *P* satisfying constraints (32) and (33). Additionally, it turns out that

(40) $$\Delta_{\min}=\min\Delta \left(P\right).$$

It is natural to consider the solutions of the identity maximization problem (39) as CP representations for contextual system S. The corresponding random variables have the highest possible degree of identity, in the presence of signaling.

The quantity

$$\Delta_{\min}-\Delta_{0}$$

is considered as the measure of “genuine contextuality”. This approach is very useful to study contextuality in the presence of signaling. The key point is the coupling of this measure of contextuality with the problem of the identity of observables measured in different contexts. As was pointed out in article [81]:

“*…contextuality means that random variables recorded under mutually incompatible conditions cannot be join together into a single system of jointly distributed random variables, provided one assumes that their identity across different conditions changes as little as possibly allowed by direct cross-influences (equivalently, by observed deviations from marginal selectivity)*”.

### 8.4. Bell–Dzhafarov–Kujala Inequality

This approach to contextuality, due to Dzhafarov–Kujala, can be reformulated in the CHSH manner by using what we can call CHSH-BDK inequality:

(41) $$\max_{ij}\left|\langle A_{11}B_{11}\rangle +\langle A_{12}B_{21}\rangle +\langle A_{21}B_{12}\rangle +\langle A_{21}B_{22}\rangle -2\langle A_{ij}B_{ji}\rangle \right|-2\Delta_{0}\leq 2.$$

It was proven that the octuple system S exhibits no genuine contextuality, i.e.,

(42) $$\Delta_{\min}=\Delta_{0},$$

if and only if the CHSH-BDK inequality is satisfied. The general formula for all cyclic systems was derived in [82], and a complete theory of cyclic systems is given in [85].

## 9. Contextuality by Default vs. Växjö Model

As was pointed out in the previous section, both CbD and the Växjö model are based on consideration of families of context labeled random variables defined on probability spaces corresponding to contexts. However, the Växjö model is endowed with the special contextual structure generating an analog of the CP calculus of conditional probabilities (see Section 2.5, Equation (4)). This leads to the contextual model of the sequential measurements and interference of probabilities. Such conditioning counterpart is not present in CbD.

The mathematical power of CbD is based on the coupling technique for CP embedding. The Växjö model also explores CP embedding, but differently from the coupling method. The context-dependent family of probability spaces $\left(P_{C}\right)$ can be embedded into a classical probability space by using the random generators for contexts selection [88,89,90,91]. This technique matches with the real experimental situation of the Bell experiments. Here the contexts correspond to the pairs of compatible observables which are selected with the aid of the random generators. In contrast to the coupling method, JPDs of random vectors $\left(A_{ij},B_{ji}\right)$ are given not as JPDs of the corresponding observables of the CP model for S, but as the classical conditional probabilities [90] (and especially [91]).

Finally, I remark that interrelation between CbD and the Växjö model model is a delicate issue. It was also analyzed in [79], with somewhat different conclusions.

## 10. No-Signaling in Quantum Theory

As was already emphasized, quantum measurement theory is free from signaling: marginals are consistent with JPDs. Now I prove this simple fact by using the Hilbert space formalism.

Consider the quantum Hilbert space formalism, a state given by density operator ρ; three observables a,b,c represented by Hermitian operators A,B,C (acting in H) with spectral families of projectors $E^{a}\left(x\right),E^{b}\left(x\right),E^{c}\left(x\right).$ It is assumed that in each pair (a,b) and (a,c), the observables are compatible; [A,B]=0,[A,C]=0. Then,

(43) $$P\left(a=x,b=y|\rho \right)=\mathrm{Tr}ӕE^{a}\left(x\right)E^{b}\left(y\right),\;P\left(a=x,c=y|ӕ\right)=\mathrm{Tr}ӕE^{a}\left(x\right)E^{c}\left(y\right)$$

and hence

(44) $$\sum_{y}P\left(a=x,b=y|\rho \right)=\mathrm{Tr}ӕE^{a}\left(x\right)\sum_{y}E^{b}\left(y\right)=\mathrm{Tr}ӕE^{a}\left(x\right)$$

$$=\mathrm{Tr}ӕE^{a}\left(x\right)\sum_{y}E^{c}\left(y\right)=\sum_{y}P\left(a=x,b=y|ӕ\right).$$

and I remark that

(45) $$\mathrm{Tr}ӕE^{a}\left(x\right)=P\left(a=x|ӕ\right)$$

and hence, both marginal probability distributions coincide with the probability of measurement of the *a*-observable alone.

I remark that this proof of no-signaling can be easily extended to generalized quantum observables given by POVMs. Thus, in quantum measurement theory there is no place for signaling. I also recall to the reader that signaling (marginal inconsistency) is absent in classical (Kolmogorov) probability theory. On the other hand, it is natural for contextual probability (as in the Växjö model).

It is useful to point out that *there is no signaling even for nonlocal quantum observables*, i.e., observables involving measurements on both counterparts of a compound system.

Now let $H=H_{1}⊗H_{2},$ where $H_{1},H_{2}$ be the state spaces of the subsystems $S_{1},S_{2}$ of the compound system $S=(S_{1},S_{2})$, and let the observables a,b,c be nonlocal, in the sense that their measurements are not localized to subsystems. The corresponding operators have the form $A=A_{1}⊗A_{2},B=B_{1}⊗B_{2},C=C_{1}⊗C_{2},$ where $A_{2},B_{1},C_{1}$ do not need to be equal to I. Let us decompose, say, $E^{a}\left(x\right)$, into tensor product $E_{1}^{a}\left(x_{1}\right)⊗E_{2}^{a}\left(x_{2}\right),$ where outcomes of *a* are labeled by pairs of numbers $(x_{1},x_{2})\to x$ (the map from pairs to the *a*-outcomes is not one to one). However, the above general scheme based on (44) is still valid. The tensor product decomposition of projections does not play any role in summation in (44).

*Nonlocality of observables cannot generate signaling.*

This is an unexpected fact, because typically signaling is associated with nonlocality. However, as we have seen, this is not nonlocality of observables.

Now we turn to the quantum CHSH inequality. As we seen in Section 6, for quantum observables, its violation is rigidly coupled only to their incompatibility. Even if $A_{i}=A_{i1}⊗A_{i2},i=1,2,$ but $[A_{1},A_{2}]=0,$ then the CHSH inequality is not violated.

Thus, by quantum theory signaling is impossible. However, e.g., in decision making, signaling patterns (expressing marginal inconsistency) were found in all known experiments.

*Should this experimental situation be considered as a contradiction between the quantum-like model for decision making and experiments?*

This is an interesting problem, and I shall analyze it in a separate paper.

Of course, we understood well that one has to distinguish theory and experiment. Even in quantum physics, no signaling corresponds to the ideal situation. One cannot completely exclude noise and other factors which might generate signaling. However, the theoretical prediction guarantees that by making experiments cleaner, one would be able to make signaling statistically insignificant. Of course, this reasoning is correct only under the assumption of validity of quantum theory for the class of systems under experimentation. Additionally, I point out that the belief in the applicability of quantum theory to cognition modeling is essentially weaker than in physics. Thus, signaling might be the irreducible feature of decision making experiments of the Bell type.

## 11. Nonconetxtual Inequalities

As before, we can consider dichotomous observables taking values ±1.

I follow the paper [41] (one of the best and clearest representations of noncontextuality inequalities). Consider a set of observables $\{x_{1},\ldots ,x_{n}\};$ contexts $C_{ij}$ determined by the pairs of indexes such that observables $x_{i},x_{j}$ are compatible—i.e., the pair $\left(x_{i},x_{j}\right)$ is jointly measurable; set $Z=\{C_{ij}\}.$ For each context $C_{ij},$ we measure correlations for observables $x_{i}$ and $x_{j}$ and averages $\langle x_{i}\rangle$ and $\langle x_{j}\rangle .$

The *n*-cycle contextuality scenario is given by the collection of contexts

(46) $$Z_{n}=\left\{\;C_{12},C_{21},\ldots ,C_{n-1,n},C_{n1}\right\}.$$

Statistical data associated with this set of contexts is given by the collection of averages and correlations:

(47) $$\{\langle x_{1}\rangle ,\ldots ,\langle x_{n}\rangle ;\langle x_{1}x_{2}\rangle ,\ldots ,\langle x_{n-1}x_{n}\rangle ,\langle x_{n}x_{1}\rangle \}.$$

Theorem 1 from paper [41] describes all tight noncontextuality inequalities. We are not interested in their general form. For n=4, we have inequality:

(48) $$|\langle x_{1}x_{2}\rangle +\langle x_{2}x_{3}\rangle +\langle x_{3}x_{4}\rangle -\langle x_{4}x_{1}\rangle |\leq 2.$$

This inequality can be rewritten in the QM notation, which we have used in the previous sections by setting $x_{1}=a_{1},x_{3}=a_{a},x_{2}=b_{1},x_{4}=b_{2}.$

Theorem 2 from article [41] demonstrates that, for

n≥4,

the aforementioned tight noncontexuality inequalities, and in particular, inequality (48), are violated by quantum correlations.

## 12. Concluding Remarks

This article was aimed at decoupling the Bell tests from the issue of nonlocality via highlighting the contextuality role. I started with discussing the physical meaning of contextuality. The common identification of contextuality with violation of the Bell type inequalities (noncontextual inequalities) cannot be accepted. This situation is illustrated by randomness theory. Here the notion of randomness is based on rigorous mathematical formalization. Statistical tests such as the NIST test are useful only to check for randomness in the outputs of random or pseudo-random generators. They are also critical to appealing to JMC, and not only because it is based on counterfactuals (cf. [151,152,153]). Here is a good place to recall that Svozil [154,155] and Griffiths [63,156,157,158] have a different viewpoint, and they suggested experimental tests for JMC. Moreover, Griffiths [156] even claimed that QM is noncontextual. Thus, the diversity of opinions about “quantum contextuality” is really amazing.

Bell considered JMC as an alternative to Einsteinian nonlocality. However, in the framework of the Bohm–Bell experiments, the physical meaning of JMC is even more mysterious than the physical meaning of EPR-nonlocality. JMC gains clear meaning only as the special case of Bohr contextuality. By the latter, outcomes of quantum observables are generated in the complex process of the interaction between a system and a measurement apparatus.

Bohr contextuality is the real seed of the complementarity principle leading to the existence of incompatible observables. This principle was also essentially clarified and demystified through connection with contextuality. My analysis led to the conclusion that contextuality and complementarity are two supplementary counterparts of one principle. It can be called the *contextuality–complementarity principle.*

This is a good place to mention the studies of Grangier, e.g., [159,160], as an attempt to suggest a heuristically natural interpretation for contextuality, which is different from JMC and Bell contextualities. Grangier contextuality is in fact also closely coupled to the Bohr complementarity principle, although this was not pointed out.

In probabilistic terms, Bohr contextuality is represented via the use of a family of Kolmogorov probability spaces which are labeled by experimental contexts. Such formalism is the *Växjö model* for contextual probability.

In this review, the problem of signaling (marginal inconsistency) was taken very seriously. We (Adenier and Khrennikov) paid attention to this problem for many years [66,67,68,69,70,71]. These publications attracted the attention of experimenters to the signaling problem. Nowadays, it is claimed that experimental data do not contain signaling patters. However, our analysis of the first loophole free Bell experiment [75] demonstrated that the statistical data suffers from signaling.

In fact, all datasets which we were able to get from experimenters and then analyze contain statistically significant signaling patters. By using induction, one may guess that even data which the owners claimed no-signaling for might suffer from signaling. Unfortunately, the author simply does not have resources to lead a new project on data analysis. Moreover, it is still difficult and often not possible at all to receive rough click-by-click data. Creation of a database for all basic quantum foundational experiments is very important for quantum foundations—starting with photo-effect and interference experiments and ending with the recent Bell-type experiments.

Can one work with statistical data shadowed by signaling? The answer to this question is positive, as was shown within the recently developed CbD theory. It led a new class of inequalities, the Bell–Dzhafarov–Kujala (BDK) inequalities. These inequalities are especially important in quantum-like studies, applications of the quantum formalism outside of physics. Presently, all experimental statistical data contain signaling patterns.

Since the incompatibility of quantum observables is mathematically encoded in the noncommutativity of corresponding operators, it is natural to try to express Bell contextuality with operator commutators. As was shown in one article [6], this is possible at least for the CHSH inequality. The basic mathematical result beyond such expression is the Landau inequality [148,149]. In the light of commutator representation of the degree of violation of the CHSH inequality, we suggest interpreting this inequality as a special test of incompatibility of observables. The commutator representation is valid for any state space; i.e., the tensor product structure does not play any role. In this way, we decouple the CHSH inequality from the problem of quantum nonlocality which was so highlighted by Bell. Incompatibility in each pair of local observables, and only incompatibility, is responsible for the inequality violation.

Finally, we studied the possible sources of signaling which are not in direct contradiction with the quantum formalism. One of such sources is disturbance in the state preparation procedure by the selection of the experimental settings. Additionally, we discussed the setting-dependent preparations in coupling to the concrete experimental situations.

## Data Availability

Not applicable.

## Appendix A. Local Realism

In quantum physics, the violation of the Bell inequalities is coupled to the violation of at least one of the following two assumptions:

(a) realism,
(b) locality.

Realism is understood as the possibility of assigning the values of observables before measurement—to consider the measurement outcome as the objective property of a system. *Bohr’s contextuality means violation of the realism assumption.* As was pointed out in Section 3.2, consideration of such contextuality is meaningful only in the presence of incompatible experimental contexts and hence incompatible observables. From the author’s viewpoint, the Bell inequality tests are designed to check for the existence of incompatible contexts and observables. The violation of these inequalities supports the Bohr complementarity principle, and hence the contextuality of quantum observables, i.e., rejection of the realism assumption. From the author’s viewpoint, nothing more can be said about the Bell tests and their foundational implications.

However, Bell highlighted the issue of nonlocality. First, the author wants to point out to ambiguity of the discussions on “quantum nonlocality”. Typically, physicists have in mind the violation of Einsteinian locality, a kind of action at a distance [42]. Therefore, experimenters separate the subsystems of a compound system as far as possible, to prevent the possibility of communications with light velocity. However, in the derivations of the Bell inequalities, the space-time structure does not appear at all. Therefore, “Bell (non)locality” has no direct coupling with Einsteinian (non)locality [161,162]. Note that the difference between the notions of Bell locality, EPR locality and nonsignaling was first specified mathematically in the article [163,164]. See also [165,166,167] for Bell locality and nonlocality. Bell locality is formulated via the introduction of hidden variables as the factorzation condition; see, e.g., [166], Equation (3). In fact, Bell nonlocality is a form of JMC expressed in terms of hidden variables, as Bell pointed out by himself [2].

This is a good place to remark that by considering the EPR–Bohm correlations in the space-time within the quantum field formalism, one finds that these correlations should decrease with distance [168,169]. The declared conservation of correlations which were apparently confirmed in the Bell experiments is the consequence of the normalization procedure used in these experiments [169].

Now we present some logical considerations:

- Local realism = realism and locality
- Not(Local realism) = Not(realism and locality) = nonrealism or nonlocality,

where “or” is the non-exclusive or operation.

The crucial point is that here, nonlocality is Bell nonlocality, not Einsteinian nonlocality. Hence, nonlocality = JMC (expressed with hidden variables). Additionally, it is a consequence of Bohr contextuality; this can also be said about nonrealism.

Thus, the whole Bell consideration can be reduced to showing that by rejecting the Bohr contextuality–complementarity principle, one can derive special inequalities for correlations. From the author’s viewpoint, the violation of these inequalities implies only that the Bohr principles hold true. Roughly speaking, one can come back to the foundations of QM which were set in the 1920s. The experimental Bell tests are advanced tests of the Bohr contextuality–complementarity principle; in this sense they are tests of quantumness.

We remark that originally, Bohr and Heisenberg appealed to the Heisenberg uncertainty relation as the basic test of incompatibility for quantum observables; e.g., [4,170,171,172] was strongly criticized, e.g., by Margenau [173] and Ballentine [95,96]. Since direct measurement of the commutator observable C=i[A,B] is difficult, the Bell tests became the most popular tests of incompatibility, and hence, quantumness. Unfortunately, the issue of incompatibility was overshadowed by “quantum nonlocality”.

## Appendix B. Kolmogorov Axiomatization of Probability

The *Kolmogorov probability space* [145] is any triple of the form

(Ω,F,P),

where Ω is a set of any origin and F is a σ-algebra of its subsets; *P* is a probability measure on F.

The set Ω represents random parameters of the model. In mathematical literature, the elements of Ω are called *elementary events*. *Events* are special sets of elementary events, those belonging to the σ-algebra F.

We remind the reader that a σ-algebra is a set system containing Ω and an empty set, closed with respect to countable unions and intersections and complements.

For example, the collection of all subsets of Ω is a σ-algebra. This σ-algebra is used in the case of finite or countable set Ω,

(A1) $$\Omega =\{\omega_{1},\ldots ,\omega_{n},\ldots \}.$$

The probability is defined as a *measure*, i.e., a map from F to non-negative real numbers which is σ-additive:

(A2) $$P\left(\cup_{j}A_{j}\right)=\sum_{j}P\left(A_{j}\right),$$

where $A_{j}\in F$ and $A_{i}\cap A_{j}=\emptyset ,i\neq j.$ The probability measure is always normalized by one:

(A3) P(Ω)=1.

In the case of a discrete probability space—see (A1)—the probability measures have the form

$$P\left(A\right)=\sum_{\omega_{j}\in A}p_{j},\;p_{j}=P\left(\left\{\omega_{j}\right\}\right).$$

A (real) random variable is a map ξ:Ω→R which is measurable with respect to the Borel σ-algebra B of R and the σ-algebra F of Ω,, i.e., for any Γ∈B, $\xi^{-1}\left(\Gamma \right)\in F.$ We recall that B is the minimal σ-algebra containing all open subsets of R (or equivalently all intervals (α,β)).

The probability distribution of a random variable ξ is defined as

$$p_{\xi }\left(\Gamma \right)=P\left(\xi^{-1}\left(\Gamma \right)\right)=P\left(\omega \in \Omega :\xi \left(\omega \right)\in \Gamma \right).$$

The mean value (average) of a random variable is defined as

(A4) $$E\left[\xi \right]=\int_{\Omega }\xi \left(\omega \right)dP\left(\omega \right)=\int_{-\infty }^{+\infty }xdp_{\xi }\left(x\right).$$

In this paper, we considered mainly random variables with the discrete range of values. For a discrete random variable ξ and its value x, we set

$$\Omega_{x}^{\xi }=\left\{\omega \in \Omega :\xi \left(\omega \right)=x\right\},$$

and the probability distribution is given by $p_{\xi }\left(x\right)=P\left(\Omega_{x}^{\xi }\right).$ The mean value has the simple representation:

(A5) $$E\left[\xi \right]=\sum_{j}\alpha_{j}p_{\xi }\left(\alpha_{j}\right).$$

In the Kolmogorov model, the conditional probability is *defined* by the *Bayes formula*

(A6) $$P\left(B|A\right)=\frac{P(B\cap A)}{P(A)},\;P\left(A\right)>0.$$

We stress that other axioms are independent of this definition.

We also present the *formula of total probability* (FTP), which is a simple consequence of the Bayes formula. Consider the pair, *a* and b, of discrete random variables. Then,

(A7) $$P\left(b=\beta \right)=\sum_{\alpha }P\left(a=\alpha \right)P\left(b=\beta |a=\alpha \right).$$

Thus the *b*-probability distribution can be calculated from the *a*-probability distribution and the conditional probabilities P(b=β|a=α). These conditional probabilities are also known as *transition probabilities.*
